# Fly transmission and mating of *Trypanosoma brucei brucei* strain 427

**DOI:** 10.1016/j.molbiopara.2008.04.009

**Published:** 2008-08

**Authors:** Lori Peacock, Vanessa Ferris, Mick Bailey, Wendy Gibson

**Affiliations:** a School of Biological Sciences, University of Bristol, Woodland Road, Bristol BS8 1UG, UK; b School of Clinical Veterinary Science, University of Bristol, Bristol BS40 7DU, UK

**Keywords:** GFP, green fluorescent protein, mRFP, modified red fluorescent protein, CM, complete Cunningham's medium, PFG, pulsed field gradient, PI, propidium iodide, VSG, variant surface glycoprotein, *Glossina morsitans*, Tsetse, Genome project, Model organism, Polyploidy

## Abstract

Like yeast, *Trypanosoma brucei* is a model organism and has a published genome sequence. Although *T. b. brucei* strain 427 is used for studies of trypanosome molecular biology, particularly antigenic variation, in many labs worldwide, this strain was not selected for the genome sequencing project as it is monomorphic and unable to complete development in the insect vector. Instead, the fly transmissible, mating competent strain TREU 927 was used for the genome project, but is not as easily grown or genetically manipulable as strain 427; furthermore, recent findings have spread concern on the potential human infectivity of TREU 927. Here we show that a 40-year-old cryopreserved line of strain 427, Variant 3, is fly transmissible and also able to undergo genetic exchange with another strain of *T. b. brucei*. Comparison of Variant 3 with lab isolates of 427 shows that all have variant surface glycoprotein genes 117, 121 and 221, and identical alleles for 3 microsatellite loci. Therefore, despite some differences in molecular karyotype, there is no doubt that Variant 3 is an ancestral line of present day 427 lab isolates. Since Variant 3 grows fast both as bloodstream forms and procyclics and is readily genetically manipulable, it may prove useful where a fly transmissible version of 427 is required.

## 1 Introduction

Like yeast, *Trypanosoma brucei* is a single-cell eukaryote used as a model organism by a number of labs worldwide. The genome sequence was recently published [1] and there have been complementary large-scale analyses of the transcriptome, proteome and functional genomics [2–4]. Much of the biochemistry and molecular biology of *T. brucei* was worked out using strain 427, a virulent lab strain originally isolated in 1960 from a sheep in Uganda and transferred to the Lister Institute in London in1961 by Keith Vickerman [5]; this strain has always been assumed to be non-human infective, e.g. [6], borne out by the lack of any report of an accidental infection over the years. Due to frequent passage, bloodstream forms of strain 427 became monomorphic, i.e. lost the ability to differentiate into short stumpy forms, a characteristic already noted in 1969 when the strain was obtained from the Lister Institute by Cross and Manning [5]. Cross subsequently used Lister 427 to derive trypanosome clones expressing a single antigen type [7]; these antigenic types are referred to as the Molteno Institute *Trypanozoon* antigen type (MITat) series and have been widely distributed to other labs.

Lister 427 is generally acknowledged to be non-tsetse transmissible. However, it can differentiate to the procyclic stage found in the tsetse midgut [8], but no further. For example, in a recent study using procyclics of a derivative clone of 427 (29–13), no salivary gland infections were found despite a high proportion of midgut infections and a prolonged sojourn in experimental flies [9]. The failure of 427 to complete the natural developmental cycle in tsetse eliminated this strain as a candidate for the *T. brucei* genome project, and another fly-transmissible strain, TREU 927, was chosen instead. However, this strain has not proved so easy to grow or as robustly genetically manipulable as 427 and recent reports have raised concerns about its potential human infectivity [10,11].

The original Lister Institute line of *T. brucei* 427 has remained cryopreserved in the trypanosome strain collection of David Godfrey, now held by the Trypanosomiasis group at the University of Bristol. Variant 3 is recorded as an 8-day variant from an infected rabbit isolated into mice on 20 March 1962. In 1992, trypanosomes from 2 capillaries of mouse blood, recorded as frozen on 20 March 1964, were passaged through mice and fly transmitted twice in succession. Here we report the results of comparison of this old line of 427, Variant 3, with more recent derivatives of Lister 427, and the results of a successful genetic cross of Variant 3 by co-transmission through tsetse with another strain of *T. b. brucei*.

## 2 Materials and methods

### 2.1 Trypanosomes

The fly transmissible trypanosome strains used were *T. b. brucei* 427 Variant 3 (MOVS/UG/60/427), referred to simply as Variant 3 in this paper, and *T. b. brucei* 1738 (MOVS/KE/70/EATRO 1738) [12]. Two non-fly-transmissible lines of 427 procyclics kindly donated by the labs of Piet Borst (Netherlands Cancer Institute) and Mark Carrington (University of Cambridge, UK) were used for comparison. For transfection, mid-log phase procyclic trypanosomes grown in Cunningham's medium [13], supplemented with 10% (v/v) heat-inactivated foetal calf serum, 5 μg/ml hemin and 10 μg/ml gentamycin (complete Cunningham's medium = CM) at 27 °C, were resuspended in Zimmerman's postfusion medium and transfected with reporter constructs by electroporation using two pulses of 1.5 kV, 25 μF [14]. Transfectants were selected 24 h post-electroporation by the addition of appropriate antibiotic (50 μg ml^−1^ hygromycin or 1 μg ml^−1^ phleomycin) and the population was checked for fluorescence by microscopy of living cells. Clones were obtained by limiting dilution of procyclics in CM in 96 well plates incubated at 27 °C in 5% CO_2_. Variant 3 was transfected with a gene for modified red fluorescent protein (mRFP) [15] targeted for integration into the tubulin locus and 1738 with a gene for enhanced green fluorescent protein (GFP) [16,17] targeted for integration into the non-transcribed spacer of the ribosomal RNA locus, on derivatives of constructs described by [18].

### 2.2 Fly transmission

Male tsetse flies from the Bristol laboratory colony of *Glossina morsitans morsitans* were kept at 25 °C and 70% relative humidity, and fed via a silicone membrane on sterile defibrinated horse blood supplemented with 2.5% (w/v) bovine serum albumin (Sigma A4503) [19] and 1 mM dATP [20]. Flies were given an infective bloodmeal as their first feed 24–48 h post-eclosion. The infective feed consisted of sterile defibrinated horse blood mixed with an aliquot of cryopreserved bloodstream form trypanosomes (approximately 10^4^ cells ml^−1^), or washed horse red blood cells mixed with *in vitro* cultured procyclics (approximately 10^7^ cells ml^−1^); infective feeds were supplemented with 60 mM *N*-acetylglucosamine to maximise infection rates [21] unless stated otherwise. For the genetic cross, the infective feed contained approximately equal numbers of cryopreserved bloodstream forms of Variant 3 mRFP and 1738 GFP; flies were dissected 36–51 days later and the midguts and salivary glands examined for infection by light and fluorescence microscopy as appropriate. Metacyclics from infected salivary glands were inoculated into mice; bloodstream forms were subsequently transformed back to procyclics by incubation in CM at 27 °C. Drug selection was carried out on these procyclic populations using hygromycin at 50 μg ml^−1^ and/or phleomycin at 1 μg ml^−1^. Trypanosomes were cloned by limiting dilution from the unselected procyclic populations.

### 2.3 Molecular analysis

Genomic DNA samples were prepared from approximately 5 × 10^7^ washed procyclics using a spin column DNA purification kit (Qiagen). Samples for pulsed field gradient (PFG) gel electrophoresis were prepared by lysing and deproteinising trypanosomes in situ in agarose blocks [22]. Microsatellite alleles were amplified by PCR as described [23] using primers PLC-G 5′ CAACGACGTTGGAAGAGTGTGAAC, PLC-H3 5′ CCACTGACCTTTCATTTGATCGCTTTC, III-2A 5′ GGTGGAATGGAAGATCAGTT, III-2B 5′ GTTGGAATTGTTGTTGCTGT, XI-53A 5′ CGTGTGTCTTGTATATCTTCT, XI-53B 5′ TGAATAAACAAAACATGAAACGAC. These 3 loci were selected on the basis of an initial screen of 1738 for allelic variation [17]. Products were resolved by electrophoresis in 1× TAE buffer through 3–5% Metaphor agarose (Cambrex) gels. Chromosomes were separated using a Biorad CHEF-DR III with a 2 phase program (Block 1: switch time 1800 s, voltage 2 V/cm, angle 106°, 15 h; Block 2: switch time 300–900 s, voltage 3 V/cm, angle 106°, 50 h) using 0.5× TBE buffer and 0.9% agarose gels. Gels were stained overnight by submersion in electrophoresis buffer containing ethidium bromide (2 μg ml^−1^) and subsequently blotted by standard methods [24]. Blots were hybridised with the following DNA fragments and washed to a stringency of 0.1× SSC at 65 °C: *VSG 221*, *VSG 121*, *VSG 117*, *β-tubulin*, *CROTI*, *KRETI*. Gene fragments were derived from cloned cDNAs (*VSG 117* [25], *VSG 121* [26], *β-tubulin* [27]), or via PCR amplification from genomic template DNA (*VSG 221*, *CROTI* and *KRETI*).

### 2.4 DNA contents

DNA contents were measured by flow cytometry on fixed and permeabilised cells stained with propidium iodide (PI) as described [17]. The laser wavelength of 488 nm excites PI but not mRFP. Background levels of staining were ascertained on unstained fluorescent and wildtype cells.

## 3 Results and discussion

### 3.1 Fly transmission

Table 1 summarizes the results of experimental fly transmission of Variant 3; the first two transmissions were made in 1992 soon after resuscitation of the original cryopreserved trypanosomes from 1964; the third transmission was carried out in 2007. In an attempt to select for fly transmissibility, metacyclic trypanosomes from each transmission were transformed into bloodstream form trypanosomes and used to infect the next batch of flies. This appears to have succeeded for transmission 2, which produced a substantial number of flies with salivary gland infection, but no further enhancement is evident for transmission 3, despite the increased rate of midgut infection brought about by supplementation of the infective feed with *N*-acetylglucosamine.

In contrast, experimental tsetse infections initiated using procyclic trypanosomes of strain 427 (obtained from the lab of Mark Carrington) produced no salivary gland infections when dissected 4–5 weeks later, although the midgut infection rate was satisfactory (41 ± 29%; *n* = 197; 4 replicates). Bloodstream form trypanosomes of a laboratory strain of 427 (obtained from the lab of Piet Borst) simply failed to differentiate in CM *in vitro* and died; bloodstream forms of fully transmissible *T. brucei* strains typically differentiate to procyclics and start to multiply in CM within 24 h.

### 3.2 Morphology and growth characteristics

**Fig. 1:**
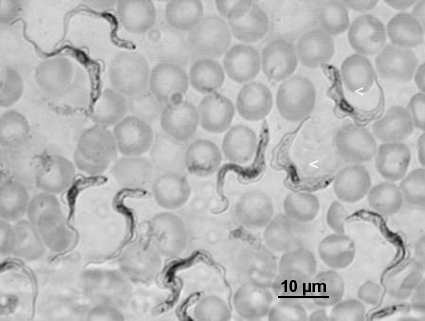
Giemsa stained bloodstream forms of Variant 3. The presence of a proportion of short-stumpy forms (arrowheads) demonstrates that the isolate is pleomorphic.

Bloodstream forms of Variant 3 grew quickly to high density in mice (population doubling time estimated as 8 h) and were noticeably faster growing than other fly transmissible *T. b. brucei* strains handled routinely in the lab such as J10 or 1738 (population doubling time estimated as 24 h). Examination of Giemsa-stained thin blood smears revealed that short-stumpy forms were present in Variant 3 (Fig. 1). Procyclics of Variant 3 also grew faster than other *T. b. brucei* strains handled routinely in the lab such as J10 or 1738 and cultures therefore withstood greater dilution ratios with fresh medium (1:10 rather than 1:5). Consequently the Variant 3 cultures were more robust during procedures such as transfection, facilitating the recovery of transfectants during drug selection.

### 3.3 Genetic cross

A red fluorescent clone of Variant 3 (Variant 3 mRFP) was mated with a green fluorescent clone of *T. b. brucei* 1738 (1738 GFP) by co-infection of tsetse flies as described previously [17,28]. Flies were dissected 36–51 days after infection and dissected organs examined for infection by phase contrast and fluorescence microscopy (Table 2). Whereas most flies examined had a mixture of red and green fluorescent trypanosomes in the midgut, only three flies had a mixed infection in the salivary glands; in two of these flies (SG2 and SG3) yellow fluorescent trypanosomes were also seen in the salivary glands. Six other flies had salivary gland infections, but these were of pure red (three) or pure green (three) trypanosomes.

Metacyclics from the three flies with mixed salivary gland infections were used to derive bloodstream forms in mice, which were subsequently transformed to procyclics *in vitro* to facilitate drug selection and cloning. Selection with hygromycin (hygromycin resistance gene linked to *GFP* gene) and/or phleomycin (phleomycin resistance gene linked to *mRFP* gene) revealed the presence of double drug resistant, i.e. hybrid, trypanosomes in the SG3 population only; these double drug resistant trypanosomes had yellow fluorescence as expected if copies of both constructs were present. The other two populations, SG1 and SG2, died out in the double drug wells; for SG1, growth was seen only with hygromycin and these trypanosomes were green as expected, while for SG2 growth was seen in wells containing the single antibiotics, with green trypanosomes in the hygromycin well and red ones in the phleomycin well as expected. Only SG3, the population in which hybrids had been unequivocally demonstrated, was analysed further.

### 3.4 Analysis of hybrid progeny

Clones were isolated from the unselected SG3 procyclic population and showed either red or yellow fluorescence; no green fluorescent trypanosomes were recovered (the hygromycin-selected well yielded only yellow trypanosomes) (Table 3). The red trypanosome clones were sensitive to hygromycin (Table 3), and the presence of neither the *GFP* nor hygromycin resistance genes could be demonstrated by PCR in these clones, whereas both genes were present in the yellow clones (not shown).

**Fig. 2:**
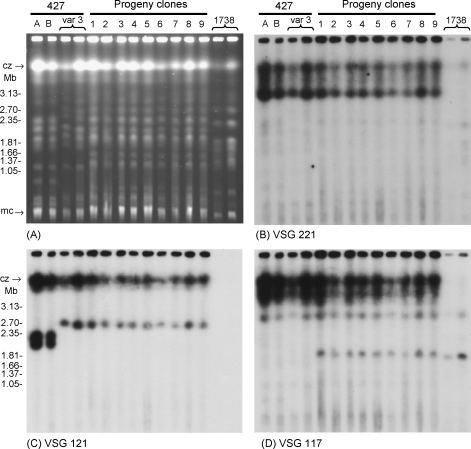
Comparison of parental and hybrid progeny clones by PFG gel electrophoresis. (A) Ethidium bromide-stained gel. From L to R: A (Amsterdam) and B (Cambridge) non-fly-transmissible laboratory lines of strain 427; var 3 = Variant 3 mRFP; progeny clones 1–9 from cross of Variant 3 mRFP × 1738 GFP; 1738 = 1738 GFP. Size marker chromosomal DNAs of *Hansenula wingei*. cz: compression zone, a region of the gel where large chromosomal DNA accumulates indiscriminately; mc: minichromosomes. (B–D) Autoradiographs of Southern blot of this gel after hybridisation with the DNA probes for three *VSG* genes as indicated. Post-hybridisational washes were to 0.1× SSC, 0.1% SDS at 65 °C.

**Fig. 3:**
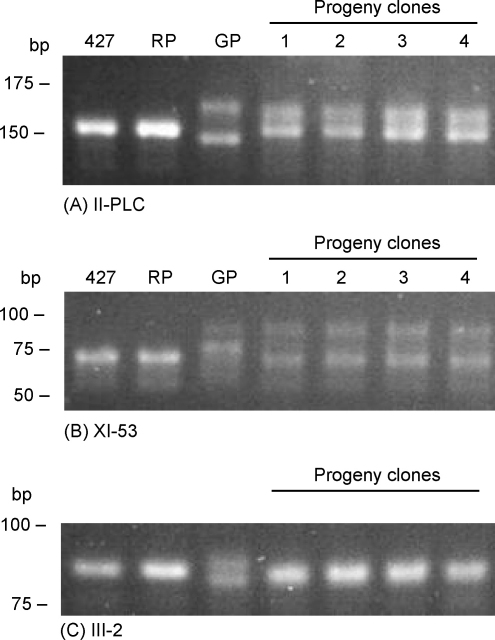
PCR amplification of microsatellite alleles. (A–C) Alleles amplified using primer sets specific for the microsatellite loci indicated, after size-separation through 4–5% Metaphor agarose gels and staining with ethidium bromide. From L to R: 427, non-fly-transmissible laboratory strain; RP, Variant 3 mRFP; GP, 1738 GFP; progeny clones 1–4.

The inheritance of chromosomal DNAs and microsatellite alleles at three loci were examined in the red and yellow progeny clones. All clones had a similar molecular karyotype, which differed from that of either parent (Fig. 2A). Chromosomal bands from both parents were present in the progeny, demonstrating that the red as well as the yellow clones were hybrids. There were also some bands of non-parental size; for example, the bands of approximately 1.2 and 1.5 Mb in the hybrid karyotypes do not exactly match any bands present in the parental karyotypes (Fig. 2A). All progeny clones were identical by analysis of microsatellite markers (Table 3). Variant 3 was homozygous for all three loci (aa), while 1738 was heterozygous (bc); each progeny clone is hybrid, with an allelic band from each parent at each of the three loci (Fig. 3).

DNA contents of the parental and progeny clones were compared by flow cytometry of PI-stained cells; results are given in Table 3. The DNA contents of the two parent clones differed markedly, Variant 3 having about 20% more DNA than 1738 (Table 3). This translates to an extra ∼10 Mb of DNA, given an estimated diploid genome size of ∼50 Mb for *T. b. brucei* based on the sequenced genome of strain TREU 927 [1]. Homologous chromosomes vary in size both within and between *T. brucei* strains [29–31], and hence it is easily possible that the extra DNA is accounted for by increased size of individual chromosomes in Variant 3. The difference in genome size between 427 and TREU 927 was previously estimated to be 16.5 Mb, representing the sum of the size differences between individual chromosomes of the two strains observed by PFG electrophoresis [31]. The extra DNA might also be accommodated by increase in size or number of other chromosomal DNAs; for example, the typical *T. brucei* genome contains an estimated 100 minichromosomes of 50–00 kb, plus a few chromosomes of intermediate size (100–800 kb), amounting to a possible 5–10 Mb of DNA in total. In addition, the kinetoplast DNA contributes to the strength of the PI fluorescence and comprises a total of 6–12 Mb of DNA, depending on how many 1 kb minicircles and 25 kb maxicircles are present.

Wherever the extra DNA is located, all nine progeny clones have substantially more DNA than either parent, ranging from 44,577 to 60,449 with a mean value of 54,169 ± 4863 (Table 3). These values are less than that predicted for the tetraploid product of fusion of the two parents, but closer to those expected for triploids resulting from fusion of haploid and diploid nuclei. Such triploid hybrid progeny have been seen in many previous trypanosome crosses [32–37,17]. Although only two alleles were observed for each clone in the microsatellite analysis (Fig. 3), more than a single allele may have been inherited from Variant 3.

### 3.5 Chromosome analysis

**Fig. 4:**
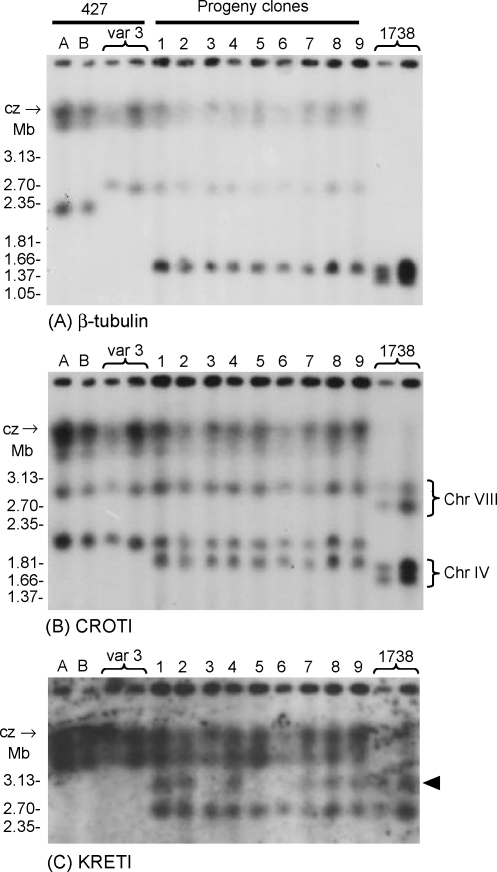
Identification of large chromosomes. (A–C) Autoradiographs of Southern blot of the PFG gel shown in Fig. 2 after hybridisation with the DNA probes for the genes indicated. Post-hybridisational washes were to 0.1× SSC, 0.1% SDS at 65 °C. Before re-use of the blot, the probe was removed by washing the blot for 10 min in 0.1 M NaOH, 0.1% SDS, followed by several changes of 2× SSC. Size marker chromosomal DNAs of *Hansenula wingei*; cz: compression zone.

Hybridisation of PFG blots with various gene probes confirmed the hybrid nature of the progeny clones (Figs. 2 and 4). The results with probes for housekeeping genes also provided evidence of aneuploidy in the hybrid clones, but did not unequivocally demonstrate triploidy for every chromosome examined (Fig. 4). The results are described in detail below.

Three *VSG* genes originally derived from Lister 427 (*VSG 117*, *VSG 121*, *VSG 221* [6]) were all present in the Variant 3 parent and also in the hybrid progeny (Fig. 2B–D); only *VSG 117* was also present in 1738, confirming previous reports that this is a well conserved gene in *T. brucei* group strains [26,38]. *VSG 221* and *VSG 121* appear to be single copy genes in Variant 3 and hybrid progeny, as there is hybridisation to a single band in each case. *VSG 221* is present on a chromosome of about 3 Mb (Fig. 2B), while *VSG 121* is present on a smaller chromosome of about 2.7 Mb (Fig. 2C); both autoradiographs also show strong hybridisation with the compression zone of the gel (arrowed in Fig. 2A), which is a region where a fraction of all the large chromosomal material accumulates. By contrast, *VSG 117* is present in at least two copies in Variant 3, as shown by hybridisation to bands of 2.8 Mb and >3 Mb (Fig. 2D). The hybrid progeny appear to have inherited these copies plus the *VSG 117* gene from 1738, which resides on a chromosomal band of about 1.9 Mb (Fig. 2D).

Hybridisation with a probe for the *β-tubulin* gene identified the two homologues of chromosome I, which carries the *tubulin* gene array (Fig. 4A). In 1738 the two homologues are quite small (1.3 Mb and 1.4 Mb), but are much larger in Variant 3 (2.6 Mb and >3 Mb), with additional hybridisation to the compression zone material; in previous analysis of the karyotype of strain 427, chromosome I homologues were estimated to be 1.85 and 3.6 Mb in size [31]. The hybrid progeny show a similar pattern of hybridisation to Variant 3, and have also inherited the 1.4 Mb homologue from 1738, suggesting that they may be trisomic for chromosome I.

Fig. 4B shows the result of hybridisation with a probe for the *CROT1* gene, which is carried on chromosomes IV and VIII [1]. In 1738 the sizes of the paired homologues are as follows: chromosome IV, 1.7 Mb and 1.8 Mb; chromosome VIII, 2.7 Mb and 2.9 Mb [17]. In Variant 3, the two homologues of chromosome IV are identical in size at approximately 2 Mb, while the chromosome VIII homologues are 2.9 Mb and >3 Mb, assuming that they have the same sizes as the chromosomes of 427 (previous estimates: chromosome IV, 1.78 and 2.3 Mb; chromosome VIII 3.4 and 3.6 Mb [31]). The hybrid progeny have chromosome IV homologues of 1.8 Mb from 1738 and 2 Mb from Variant 3, and chromosome VIII homologues of 2.9 Mb either from 1738 or Variant 3, and of >3 Mb from Variant 3. There is no evidence of trisomy for chromosome IV in the hybrid progeny, since the intensity of the 1.8 Mb and 2 Mb bands is equal. This is harder to judge for chromosome VIII, because of trapping of material in the compression zone and the non-equivalent hybridisation of the two homologues in 1738.

Fig. 4C shows the result of hybridisation with a probe for the *KRETI* gene, which is carried on chromosome VII [1]. In 1738 the sizes of the homologues are 2.6 Mb and 3 Mb; previous results showed that the *GFP* gene had integrated into an array of ribosomal RNA genes on the smaller homologue of chromosome VII in 1738, but had frequently switched to the larger homologue in hybrid progeny [17]. In Variant 3, hybridisation is to a band of >3 Mb and the compression zone; in previous analysis of the karyotype of strain 427, both chromosome VII homologues were identical in size, estimated to be 3.4 Mb [31]. All the progeny clones show a similar pattern of hybridisation to Variant 3, and have also inherited one (clones 3, 5 and 6) or both (clones 1, 2, 4, 7–9) of the 1738 homologues. This is the only hybridisation result that reveals any difference between the 9 progeny clones or that shows inheritance of more than 1 homologue from 1738. The presence of the 3 Mb band also distinguishes clone 1 from the other red fluorescent hybrid clones (3, 5, and 6). All these clones would be expected to carry the *GFP* gene on the 2.6 Mb chromosome VII homologue of parent 1738, and its absence from this homologue in clones 3, 5, and 6, and from both homologues in clone 1, indicates that it has been lost, presumably by homologous recombination or gene conversion.

In summary, all nine progeny clones recovered from fly SG3 were hybrids with a DNA content of approximately 3N relative to the parental trypanosomes. The clones divide into three groups by phenotype and genotype: clone 1; clones 3, 5, 6; clones 2, 4, 7–9 (Table 3).

### 3.6 Comparison of Variant 3 with lister 427

The identity of Variant 3 as a fly transmissible ancestor in the Lister 427 lineage was examined by comparison of the karyotype and microsatellite alleles of Variant 3 with two non-fly transmissible laboratory lines of Lister 427. *VSG 221*, a VSG gene so far found only in 427 [26], is present in the same location in Variant 3 and 427 lines A and B (Fig. 2B), and the patterns of hybridisation with *VSG 117*, *CROTI* and *KRETI* are also identical (Fig. 2D, Fig. 4B and C). In addition, analysis of three microsatellite markers showed Variant 3 to be identical to the 427 lines (Fig. 3). At each locus, the three strains are homozygous. Although there is no published comparative data for these three microsatellite loci, microsatellites are generally found to be hypervariable markers for population genetics analysis and we would therefore expect allele size to be variable among *T. brucei* sspp. strains. With a conservative estimate of ten different alleles at each locus, the probability of finding two identical but unrelated strains would be 10^−6^.

On the other hand, while the two laboratory lines of 427 have identical karyotypes, the karyotype of Variant 3 shows some differences. For example, Variant 3 lacks the small chromosomal band of about 0.8 Mb present in the laboratory lines, and has an extra band of about 2.7 Mb. Fig. 2C shows that the location of *VSG 121* is clearly different, with at least two copies of the gene in the 427 lines. One copy of *VSG 121* co-localizes with *β-tubulin* (compare Fig. 2C and Fig. 4A), indicating that it is on the smaller homologue of chromosome I in Variant 3 and the 427 lines, despite the size change in this homologue (2.2 Mb in 427 lines A and B, 2.6 Mb in Variant 3; Fig. 4A). This is in agreement with detailed analysis of the 427 karyotype where copies of the *VSG 121* gene were found on chromosomes I and IV [31].

On balance, while Variant 3 is not identical to the laboratory lines of 427, it is very similar, and the observed chromosomal size differences could well be the result of chromosomal recombination during long-term laboratory passage and selection of particular lines.

## 4 Conclusion

Variant 3, which was cryopreserved over 40 years ago, indisputably belongs to the lineage of Lister 427 in current widespread laboratory use. In contrast to these laboratory isolates, Variant 3 is tsetse transmissible and we demonstrate here that it is also capable of genetic exchange. Notwithstanding, Variant 3 shares the Lister 427 characteristics of robust growth and easy handling, which have made Lister 427 a favourite laboratory strain.

All the progeny clones from the genetic cross of Variant 3 with another *T. b. brucei* strain had DNA contents consistent with 3N, and PFG analysis showed that more than the normal two copies of some of the large chromosomes were present. While hybrids with raised DNA contents have been recovered in other *T. b. brucei* crosses, the fact that no diploid progeny were recovered in this cross suggests a severe problem. One possibility is strain incompatibility due to the DNA content of Variant 3 being 20% greater than that of its mate, 1738. We still do not know the exact details of the process of genetic exchange in trypanosomes, although there is evidence that both meiosis and cell fusion take place. If parental nuclei fuse before meiosis in *T. brucei*, then it is possible that there were problems in meiotic pairing between the larger chromosomes of Variant 3 and their homologues from 1738 [39]. Alternatively, if meiosis precedes fusion, 1738 is unlikely to be at fault, since the majority of hybrid progeny from a previous cross of 1738 with another similar *T. b. brucei* strain, J10, were diploid [17]; rather, the problem lies with Variant 3 and perhaps it is unable to undergo meiosis properly. However, the results do not fit with the hypothesis that fusion of haploid 1738 and diploid Variant 3 gametes occurred, as both homologues of chromosome VII from 1738 were present in some of the hybrid progeny. This implies that the normal process of meiosis has been disrupted in both 1738 and Variant 3, perhaps through faulty signalling between the two cells or through problems with their cellular or chromosomal interactions. Interestingly, an exactly analogous result was reported in a previous cross, in which the two parental trypanosomes differed in DNA content, STIB 386 having about 30% more DNA than TREU 927 [37]; hybrids with DNA contents consistent with both 2N and 3N were recovered from this cross and analysis of the 3N hybrid genotype mostly supported the hypothesis of fusion of a haploid genome of TREU 927 with a diploid genome of STIB 386, except for chromosome V, for which there were two TREU 927 homologues present and only one from STIB 386 [37]. As for the cross described here, this suggests simultaneous errors during meiotic division for both parental trypanosomes. Further crosses of Variant 3 with other, more similar strains of *T. b. brucei* may help unravel this puzzle.

If Variant 3 is the wildtype of Lister 427, then current laboratory isolates can be regarded as developmentally defective mutants and comparison of the mutant and wildtype strains should provide information on the genetic control of development.

## Figures and Tables

**Table 1:** Tsetse transmission of Variant 3

Transmission	Duration of infection (days)	Midgut infection rate	Salivary gland infection rate	Transmission index
1	35	32/80 (40%)^a^	1/80 (1%)	1/32 (3%)
2	56	27/82 (33%)^a^	7/82 (9%)	7/27 (26%)
3	29–41	99/132 (75%)	6/132 (5%)	6/99 (6%)

a Infective feeds not supplemented with *N*-acetylglucosamine.

**Table 2:** Experimental cross of Variant 3 mRFP with 1738 GFP

Number of flies with infected midguts (%)	110/196 (56%)
Number of flies with infected salivary glands (%)	9/196 (5%)
Number of flies with mixture of red/green trypanosomes in midgut (%)	32/34 (94%)
Number of flies with mixture of red/green trypanosomes in salivary glands (%)	3/9 (33%)^a^

a Yellow trypanosomes were seen in salivary glands from two flies with a mixed infection.

**Table 3:** Cloned progeny from cross of Variant 3 mRFP and 1738 GFP

Clone	Fluorescence	Drug resistance^a^	Molecular karyotype	Microsatellite profile			DNA content^b^
				II-PLC	XI-53	III-2	
Variant 3 mRFP	Red	Phleo	427	aa	aa	aa	35,422
1738 GFP	Green	Hyg	1738	bc	bc	bc	28,932
1	Red	Phleo	427/1738	ac	ac	ab	54,155
2	Yellow	Phleo/Hyg	427/1738	ac	ac	ab	53,549
3	Red	Phleo	427/1738	ac	ac	ab	54,701
4	Yellow	Phleo/Hyg	427/1738	ac	ac	ab	56,541
5	Red	Phleo	427/1738	ac	ac	ab	60,296
6	Red	Phleo	427/1738	ac	ac	ab	60,449
7	Yellow	Phleo/Hyg	427/1738	ac	ac	ab	52,310
8	Yellow	Phleo/Hyg	427/1738	ac	ac	ab	44,577
9	Yellow	Phleo/Hyg	427/1738	ac	ac	ab	50,941

a Phleo, phleomycin; Hyg, hygromycin.

b Mean value for red fluorescence in arbitrary units for G1 peak of flow cytometry histogram.
